# Sphingosine Kinase 1 Regulates the Akt/FOXO3a/Bim Pathway and Contributes to Apoptosis Resistance in Glioma Cells

**DOI:** 10.1371/journal.pone.0019946

**Published:** 2011-05-18

**Authors:** Hongyu Guan, Libing Song, Junchao Cai, Yongbo Huang, Jueheng Wu, Jie Yuan, Jun Li, Mengfeng Li

**Affiliations:** 1 Department of Endocrinology and Diabetes Center, The First Affiliated Hospital of Sun Yat-sen University, Guangzhou, Guangdong, China; 2 State Key Laboratory of Oncology in Southern China, Sun Yat-Sen University Cancer Center, Guangzhou, Guangdong, China; 3 Key Laboratory of Tropical Disease Control (Sun Yat-Sen University), Ministry of Education, Guangzhou, Guangdong, China; 4 Department of Microbiology, Zhongshan School of Medicine, Sun Yat-Sen University, Guangzhou, Guangdong, China; 5 Key Laboratory of Functional Molecules from Oceanic Microorganisms (Sun Yat-sen University), Department of Education of Guangdong Province, Guangzhou, China; 6 Department of Biochemistry, Zhongshan School of Medicine, Sun Yat-Sen University, Guangzhou, Guangdong, China; University of California, Los Angeles, and Cedars-Sinai Medical Center, United States of America

## Abstract

The aim of this study was to investigate the mechanism through which Sphingosine kinase-1 (SPHK1) exerts its anti-apoptosis activity in glioma cancer cells. We here report that dysregulation of SPHK1 alters the sensitivity of glioma to apoptosis both *in vitro* and *in vivo*. Further mechanistic study examined the expression of Bcl-2 family members, including Bcl-2, Mcl-1, Bax and Bim, in SPHK1-overexpressing glioma cells and revealed that only pro-apoptotic Bim was downregulated by SPHK1. Moreover, the transcriptional level of Bim was also altered by SPHK1 in glioma cells. We next confirmed the correlation between SPHK1 and Bim expression in primary glioma specimens. Importantly, increasing SPHK1 expression in glioma cells markedly elevated Akt activity and phosphorylated inactivation of FOXO3a, which led to downregulation of Bim. A pharmacological approach showed that these effects of SPHK1 were dependent on phosphatidylinositol 3-kinase (PI3K). Furthermore, effects of SPHK1 on Akt/FOXO3a/Bim pathway could be reversed by SPHK1 specific RNA interference or SPHK1 inhibitor. Collectively, our results indicate that regulation of the Akt/FOXO3a/Bim pathway may be a novel mechanism by which SPHK1 protects glioma cells from apoptosis, thereby involved in glioma tumorigenesis.

## Introduction

Glioma is the most common form of primary brain tumors [1]. The low overall survival rate of glioma patients has remained unchanged over decades despite that many efforts have been made in improving the treatment of glioma. Impairments in apoptotic programs are tightly linked to the failure of clinical cancer therapy, such as chemotherapy and radiotherapy, and thus, rational development of more efficacious anti-glioma therapies will largely rely on revealing mechanisms involved in regulating the apoptotic programs in glioma cells [2]–[4].

SPHK1, an oncogenic enzyme, has been found to induce a transforming phenotype in fibroblasts and tumor formation in nude mice [5], [6]. It was also found that in a variety of tumor types, including glioma, SPHK1 mRNA and protein were significantly elevated [7]–[10]. The oncogenic function of SPHK1 has been linked to deregulated apoptotic pathways, as ectopic expression of SPHK1 could promote cell proliferation and induce resistance to apoptosis [11]. The molecular mechanism underlying the above oncogenic features of SPHK1, however, remains largely unknown.

Forkhead box O (FOXO) transcription factors are a subfamily of forkhead proteins characterized by a ‘winged-helix’ DNA-binding domain termed the forkhead box [12]. It has been documented that members of the FOXO subfamily are involved in various signaling pathways and modulate a wide range of biochemical processes, such as cell cycle progression, differentiation, DNA damage repair, and programmed cell death [13]. FOXO family proteins regulate cell survival by transcriptionally modulating the expression of death receptor ligands and Bim, a pro-apoptotic protein belongs to the BH3-only subgroup of BCL-2 family [14], [15]. The transcriptional activity of FOXO3a is regulated by the phosphorylation status mediated by Akt kinase, which has been shown to be activated in a variety of cancer types, including gliomas [16]–[18]. Upon stimulation of the PI3K/Akt signaling cascade, the phosphorylated FOXO3a proteins bind to the 14-3-3 proteins in the nucleus and are subsequently exported from the nucleus immediately, thereby inhibiting FOXO3a-dependent transcription. Conversely, inactivation of Akt activity results in FOXO3a dephosphorylation, nuclear translocation, and enhanced transcription of target genes [15]. Interestingly, it has been demonstrated that upregulation of SPHK1 could induce resistance to apoptosis by a mechanism involving activation of the PI3K/Akt pathway in HS1 erythroleukemic cells [19].

In the current study, we report that SPHK1 overexpression suppressed apoptosis induced by UV radiation or adriamycin via downregulation of Bim. Such effects of SPHK1 were further confirmed by shRNA-mediated knockdown experiments. Furthermore, we demonstrated that downregulation of Bim was mediated by Akt activation, which subsequently induced phosphorylation and inactivation of FOXO3a in glioma cells. Taken together, our results suggest that the PI3K/Akt/FOXO3a/Bim axis might constitute a signaling cascade that mediates the anti-apoptotic activity of SPHK1 in glioma cells and thereby play an essential role in the development and progression of glioma, as well as in its insensitivity to pro-apoptotic therapies.

## Materials and Methods

### Ethics Statement

#### Glioma tissues

A total of 82 paraffin-embedded glioma samples, which had been clinically and histologically diagnosed at the First Affiliated Hospital of Sun Yat-sen University during year of 2000 to 2002, were obtained with prior written informed consents from the patients and approval from the Institutional Research Ethics Committees of Sun Yat-sen University and its First Affiliated Hospital. Clinical and clinicopathologic classification and staging were determined according to the World Health Organization (WHO) [20]. Clinical information on the samples is summarized as described previously by us, including 11 cases of WHO grade I, 21 cases of WHO grade II, 30 cases of WHO grade III, and 20 cases of WHO grade IV tumors.

### Cell lines and treatments

Glioma cell lines U87MG and LN-382 were kindly provided by Dr. Shi-Yuan Cheng at the Department of Pathology, University of Pittsburg, and maintained in DMEM supplemented with 10% FBS [21]. In some experiments, adriamycin (Sigma, St. Louis, MO), LY294002 (Cell Signaling, Beverly, MA), or sphingosine kinase inhibitor (2-(p-Hydroxyanilino)-4-(p-chlorophenyl) thiazole, SK-I) (Calbiochem, La Jolla, CA) dissolved in dimethyl sulfoxide (DMSO), was used to treat cells at indicated final concentrations and times.

### Vectors and retroviral infection

SPHK1 construct was generated by sub-cloning PCR- amplified full-length human SPHK1 cDNA into pMSCV. For depletion of SPHK1, two human siRNA sequences were cloned into pSuper-retro-puro to generate pSuper-retro-SPHK1-RNAi(s), respectively, and the sequences are RNAi#1: GGCTGAA ATCTCCTTCACG; RNAi#2: GGGCAAGGCCTTGCAGCTC. Retroviral production and infection were performed as described previously [21], [22].

### Western blotting (WB)

Western blotting was performed according to standard methods as described previously [20]. The following primary antibodies were used: anti-SPHK1 (Abcam, Cambridge, MA), anti-poly ADP-ribose polymerase (PARP), anti-caspase-3, anti-Bcl-2, anti-Mcl-1, anti-Bax, anti-Bim, , anti-FOXO3a anti-phospho-FOXO3a (Ser^253^), anti-phospho-Akt (Thr^308^), anti-phospho-Akt (Ser^473^), anti-total Akt (Cell Signaling, Beverly, MA), and anti-α-tubulin (Sigma-Aldrich, St Louis, MO). The nuclear extracts were prepared using the Nuclear Extraction kit (Active Motif, Tokyo, Japan) as the manufacturer instructed.

### Sphingosine Kinase Activity Assay

The activity of sphingosine kinase was quantified by using a commercial Sphingosine Kinase Activity Assay Kit (Echelon Biosciences, Salt Lake City, UT) as the manufacturer instructed.

### Cell survival assay

Cells were plated in six-well plates at density of 1×10^5^ cells/well and incubated for 24 h, followed by adriamycin treatment (0.5, 1.0 or 2.0 µM) or irradiation with UV_245 nm_ (20, 40, 60 J/m^2^) and further incubation for 72 h. Numbers of surviving cells were determined by the trypan blue exclusion method.

### TUNEL assay

The DeadEnd™ Fluorometric TUNEL System (Promega, Madison, WI) was used for TUNEL assay according to the manufacturer's instruction.

### Annexin-V binding assay

The ApopNexin™ FITC Apoptosis Detection Kit (Millipore, Lake Placid, NY) was used to quantitatively examine apoptotic cells according to manufacturer's instruction.

### Subcutaneous xenograft mouse model

Animal protocols were approved by the Ethical Committee of Sun Yat-sen University. Nude mice were injected s.c. with 2×10^6^ cells (U87MG/vector, U87MG/SPHK1, U87MG/RNAi vector and U87MG-SPHK1-RNAi) in 0.1 mL of PBS in the right flank. When the tumor size reached approximately 50 mm^3^, the mice were injected i.p. every 3 d with 5 mg/kg epirubicin (4′-epidoxorubicin, Pfizer, New York, NY), an antineoplastic agent derived from adriamycin, dissolved in saline. In another set of experiment, nude mice were injected s.c. with 2×10^6^ cells (U87MG-vector) in 0.1 mL of PBS in the right flank. When the tumor size reached to 50 mm^3^, the mice were randomized into two groups (n = 5) and injected intraperitoneally either with epirubicin (5 mg/kg) or epirubicin(5 mg/kg) plus SK1-I (50 mg/kg) every 3 days, up to 15days. Tumor volumes were measured with caliper and calculated using formula: 0.52×(length in millimeters)×(width in millimeters) ^2^. At the end of the experiment, tumors were excised, fixed in formalin and embedded in paraffin.

### RNA extraction and real-time RT-PCR

RNA extraction, RT and real-time PCR were performed as described previously [9]. The primers selected are as the following: Bim, forward: 5′-TATGAGAAG ATCCTCCCTGC-3′ and reverse: 5′-ATATCTGCAGGTTCAGCC TG-3′; GAPDH, forward: 5′-ACCACAGTCCATGCCATCAC-3′ and reverse: 5′-TCCACCACCCTG TTGCTGTA-3′.

### Luciferase activity assay

Dual-Luciferase reporter assays were performed according to the manufacturer's instructions (Promega, Madison, WI). Briefly, 5×10^4^ cells were seeded in triplicates in 24-well plates and allowed to settle for 24 h. Two hundred nanograms of pGL3-3×IRS plasmid or control-luciferase plasmid plus 10 ng pRL-TK renilla plasmid were transfected into the cells using the Lipofectamine 2000 reagent (Invitrogen Co., Carlsbad, CA). Luciferase and Renilla luciferase activities were determined 48 h after transfection for each sample according to the manufacturer's instruction. The reporter plasmid for quantitatively detecting the transcriptional activity of FoxO3a was generated essentially as described [23]. Briefly, oligonucleotides containing an array of three copies of an insulin response sequence (IRS) (5′-GCAAAACAAACTTATTTTGAAGCAAAACAAACTTAT TTTGAAGCAAA ACAAACT TATTTTGAA-3′ and 5′-AGCTTTCAAAATAAGTTTGTT TTGCTTCAAAA TAAGTTTGTTT TGCTTCAAAATAAGTTTGTTTTGCGTAC -3′) were annealed to form a double-stranded oligonucleotide and ligated into the *Kpn* I and *Hind* III sites of the pGL3-Enhancer plasmid (Promega, Madison, WI) to create pGL3-3×IRS.

### Immunohistochemistry

Immunohistochemical (IHC) analysis was performed to study altered protein expression in human glioma tissue. The scores of SPHK1 and Bim were carried out similarly to previously described methods [9]. In brief, the degree of immunostaining of SPHK1 and Bim proteins were evaluated and scored independently by two observers, considering both the proportion of positive staining tumor cells and the staining intensity. Scores representing the proportion of positive stained tumor cells was graded as: 0 (no positive tumor cells), 1 (<10%), 2 (10–50%), and 3 (>50%). The intensity of staining was determined as: 0 (no staining); 1 (weak staining = light yellow), 2 (moderate staining = yellow brown), and 3 (strong staining = brown). The staining index (SI) was calculated as the product of staining intensity and percentage of positive tumor cells, resulting in scores as 0, 1, 2, 3, 4, 6 and 9. Cutoff values for SPHK1 were chosen based on a measurement of heterogeneity using the log-rank test with respect to overall survival. We identified the optimal cutoff as: the SI score of ≥4 was considered as high expression, and ≤3 as low expression.

### SiRNA

FOXO3a specific siRNA oligo was purchased from Ribobio (Guangzhou, China). The sense sequence is 5′-GAGCTCTTGGTGGATCATC-3′. SiRNA was transfected into cells cultured in 6-well plates using Lipofectamine 2000 following the manufacturer's instructions (Invitrogen, Carlsbad, CA). Following transfection with siRNA, cells were cultured for 2 days before use.

### Statistical analysis

All statistical analyses were carried out using the SPSS 11.0 statistical software package. Bivariate correlation between SPHK1 and Bim expression were calculated by Spearmans rank correlation coefficients. All values represent at least three independent experiments and are expressed as the means ± standard deviation (SD). The differences between experimental conditions were compared on a one-to-one basis using Student's *t* tests. *p*<0.05 was considered statistically significant.

## Results

### Dysregulation of SPHK1 altered the apoptotic sensitivity of glioma cells both *in vitro* and *in vivo*

**Figure 1:**
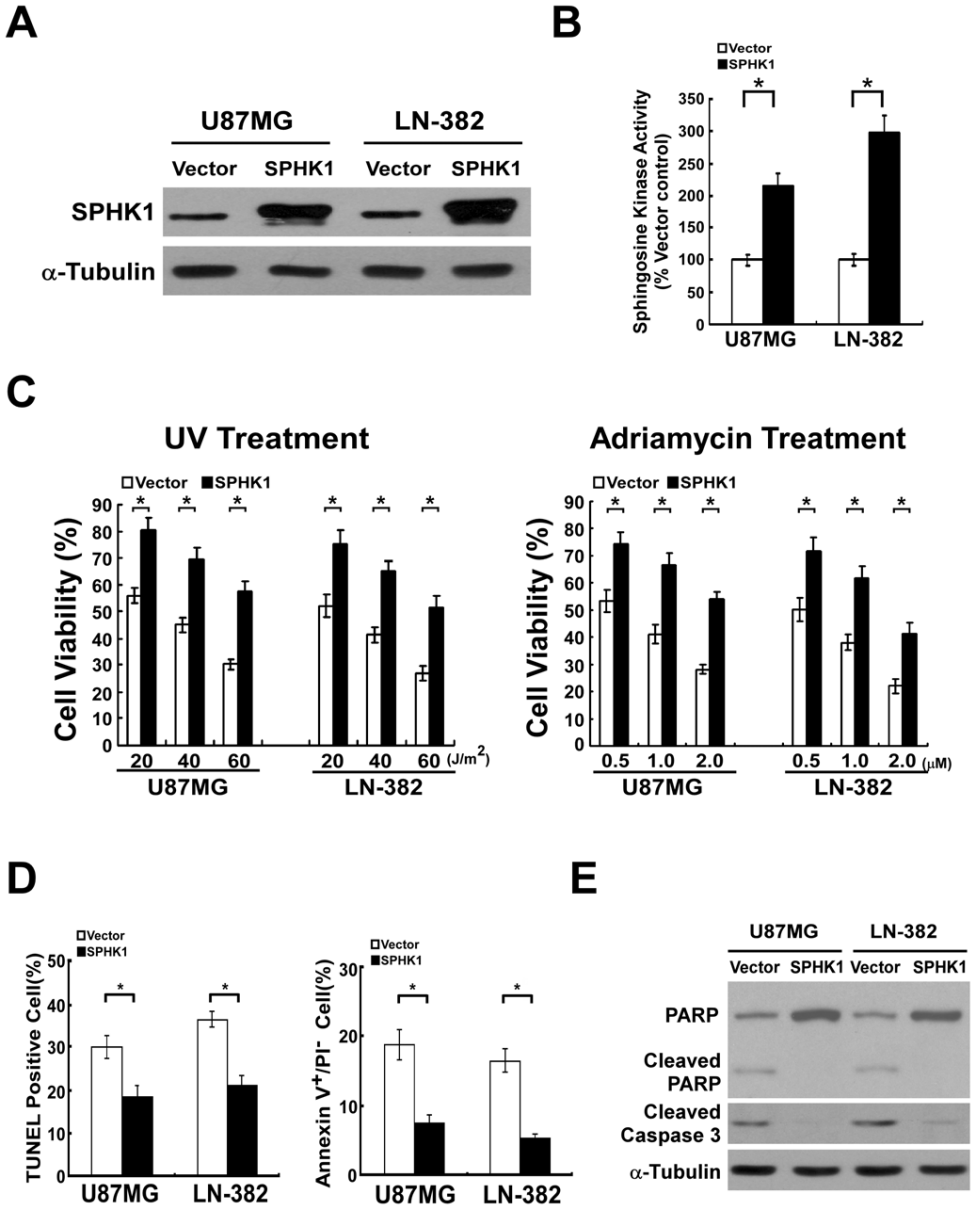
Glioma cells overexpressing SPHK1 protein are less sensitive to UV- or adriamycin-induced apoptosis. (A) Overexpression of SPHK1 in glioma cell lines analyzed by WB. α-tubulin was used as a loading control. (B) SPHK1 enzymatic activity in SPHK1-overexpressed glioma cells was markedly increased. (C) Overexpression of SPHK1 inhibited cell death induced by adriamycin (left panel) or UV irradiation (right panel). Seventy-two hours after treatment with adriamycin or UV irradiation, cell viability was assessed by the trypan blue exclusion method. (D) SPHK1 prevented adriamycin-induced apoptosis of glioma cells. Annexin-V binding and TUNEL assays of indicated cells were performed after incubation with adriamycin for 6 h and 24 h, respectively. Quantification of TUNEL positive cells (left panel) and Annexin V^+^/PI^−^ cells (right panel). Results are expressed as percentages of total cells. (E) PARP cleavage and cleaved caspase 3 levels were assessed in indicated cells treated with UV irradiation (40 J/m^2^) or adriamycin (1.0 µM) for 24 h via WB. α-tubulin was used as a loading control. For (B), (C) and (D), error bars represent mean ± SD from three independent experiments with similar results. *, *p*<0.05.

**Figure 2:**
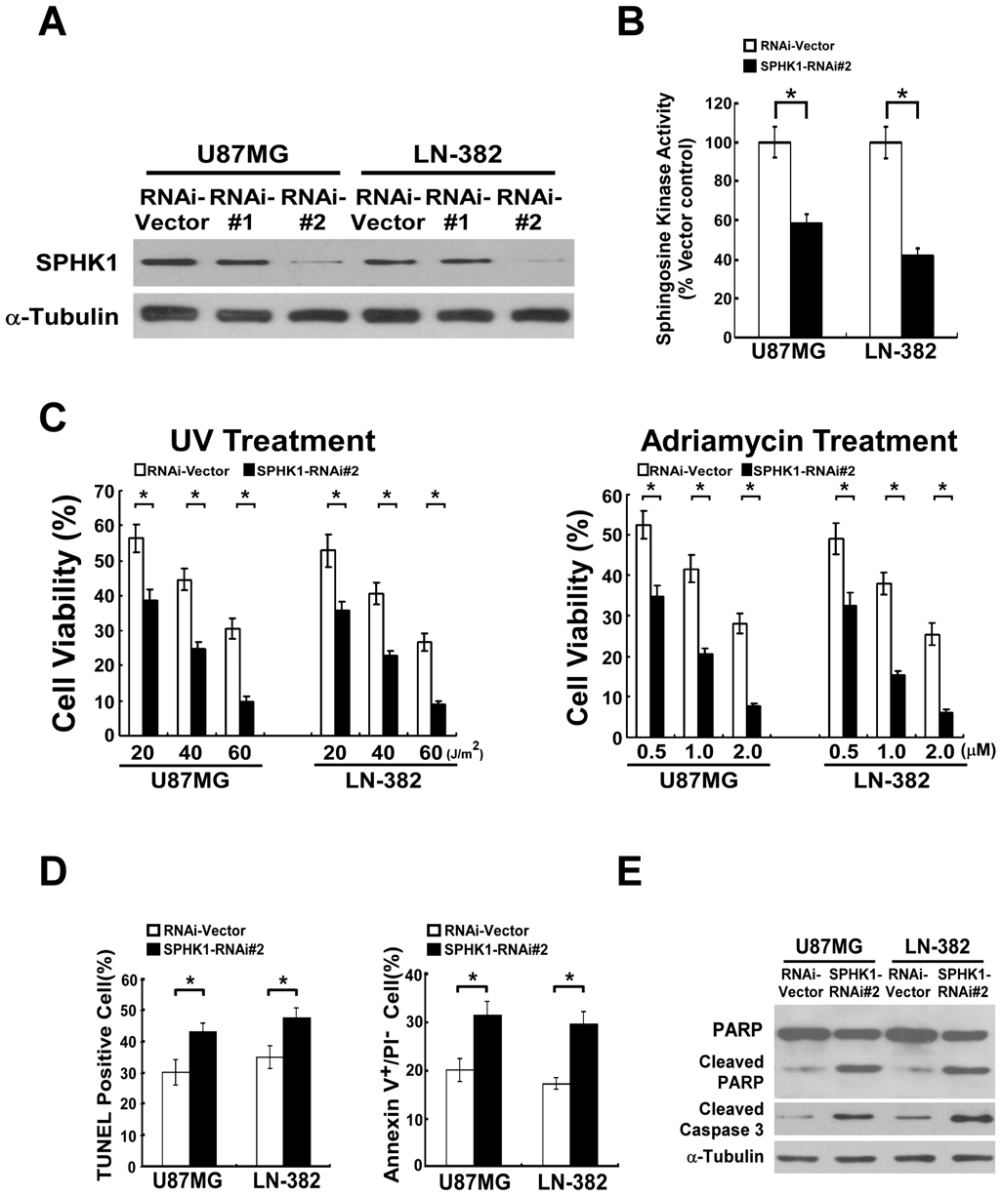
Downregulation of SPHK1 expression decreases the resistance of glioma cells to apoptosis. (A) SPHK1 knockdown was achieved by introducing specific shRNAs in glioma cells. α-tubulin was used as a loading control. (B) Silencing of SPHK1 led to significant decrease of enzymatic activity in glioma cells. (C) Knockdown of SPHK1 enhances cell death. (D) SPHK1 knockdown enhanced the sensitivity of glioma cells to adriamycin-induced apoptosis. Quantification of TUNEL positive cells (left panel) and Annexin V^+^/PI^−^ cells (right panel). Results are expressed as percentages of total cells. (E) PARP cleavage and cleaved caspase 3 levels were assessed in indicated cell lines treated with UV irradiation (40 J/m^2^) or adriamycin (1.0 µM) for 24 h via WB. α-tubulin was used as a loading control. For (B), (C) and (D), error bars represent mean ± SD from three independent experiments with similar results. *, *p*<0.05.

To determine whether dysregulation of SPHK1 in glioma cells could alter resistance to apoptosis, stable glioma cell lines (U87MG and LN-382) with SPHK1 overexpression or knockdown were established (Figures 1A and 2A). The SPHK1 enzymatic activities in SPHK1 overexpressed glioma cells were markedly increased in comparison to that in vector-control cells, whereas silencing of SPHK1 led to significant decrease of SPHK1 activity (Figures 1B and 2B). As expected, both SPHK1-expressing cell lines showed stronger resistance to treatment of various doses of UV irradiation and adriamycin in comparison with the vector-control, which indicated that overexpression of SPHK1 could significantly reduced cell death induced by UV irradiation and chemotherapeutic drug adriamycin (Figures 1C). Furthermore, TUNEL and Annexin-V binding assays were performed to assess that the increased viability of SPHK1 overexpressing cells was due to apoptosis inhibition. As shown in Figure 1D, the number of apoptotic cells in vector-infected cells was significantly higher than that in SPHK1-overexpressing cells upon 1.0 µM adriamycin treatment, suggesting that ectopic expression of SPHK1 protected glioma cells from pro-apoptotic induction. Moreover, we characterized the effect of SPHK1 on apoptotic protection by examining the cleavages of pro-caspase 3 and PARP in glioma cells expressing SPHK1. As shown in Figure 1E, cleavages of both caspase 3 and PARP were suppressed in the SPHK1-overexpressing cells. In contrast, silencing SPHK1 by RNAi#2, which showed markedly decreased expression of SPHK1, dramatically enhanced UV- or adriamycin-induced cell death in glioma cells (Figures 2C). Results of TUNEL and Annexin-V binding assays indicated that the decreased viability of SPHK1-downregulated cells was due to increased apoptosis susceptibility (Figure 2D). In addition, activating cleavages of PARP and caspase 3 have been shown to be markedly increased in SPHK1-downregulated cells (Figure 2E). Taken together, these data suggest that SPHK1 acts to stimulate survival and anti-apoptotic signaling in glioma cells.

**Figure 3:**
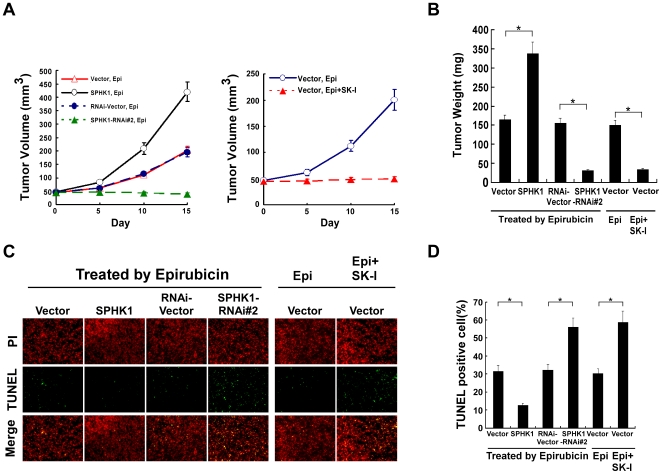
The effects of dysregulation of SPHK1 expression on epirubicin induced-apoptosis *in vivo*. (A) Tumor volumes were measured on the indicated days. Left panel, indicated cells (2×10^6^) were injected in the flank of nude mice. When the mean tumor volume reached approximately 50 mm^3^, the mice were injected i.p. with epirubicin (5 mg/kg), every 3 days, up to 15 days. Right panel, U87MG-vector cells (2×10^6^) were injected in the flank of nude mice. When the mean tumor volume reached approximately 50 mm^3^, the mice were randomized into two groups (n = 5) and injected intraperitoneally either with epirubicin (Epi, 5 mg/kg) or epirubicin (Epi, 5 mg/kg) plus SK1-I (50 mg/kg) every 3 days, up to 15days. (B) Mean tumor weights were measured. Immunofluorescent images (C) and quantification (D) of TUNEL positive cells. For (B) and (D), error bars represent mean ± SD from three independent experiments with similar results *, *p*<0.05.

To further confirm the anti-apoptotic potential of SPHK1 *in vivo*, we decided to examine whether dysregulation of SPHK1 could alter the sensitivity of glioma cells to apoptosis in nude mice. When the volume of tumors reached approximately 50 mm^3^, the nude mice were injected intraperitoneally with the adriamycin analogue epirubicin (5 mg/kg) every 3 days, up to 15days. As shown in Figures 3A and 3B, the U87MG-SPHK1 cells formed tumors and displaying larger tumor sizes and higher tumor weights than those formed by the U87MG-vector control cells. Whereas, the tumors formed from U87MG-SPHK1-RNAi cells were smaller, in size and weight, than those of vector control cell-formed tumors. Furthermore, TUNEL assay indicated that the percentage of apoptotic cells in U87MG control cell-formed tumors was significantly higher than that in the tumors formed from U87MG-SPHK1 cells, but lower than that of the U87MG-SPHK1-RNAi cell-formed tumors (Figures 3C and 3D). Moreover, the impact of SPHK1 inhibition was examined, using a specific SPHK1 inhibitor (SK1-I), on the resistance of gliomas to epirubicin treatment. When U87MG control cells-formed tumors reached a mean volume of approximately 50∼100 mm^3^, the nude mice were randomized into two groups (n = 5) and injected intraperitoneally either with epirubicin (5 mg/kg) or epirubicin (5 mg/kg) plus SK1-I (50 mg/kg) every 3 days, up to 15days. As compared with epirubicin treated group, both the tumor sizes and tumor weights in mice treated by epirubicin plus SK1-I were dramatically reduced (Figures 3A and 3B). TUNEL assay revealed that inhibition of SPHK1 activity enhanced epirubicin-induced apoptosis of glioma cells (Figures 3C and 3D). Taken together, our results indicate that up-regulation of SPHK1 enhanced the resistance of glioma cells to cytotoxic reagent-induced cell apoptosis, and targeting SPHK1 through inhibition of SPHK1 activity might represent a potential therapeutical strategy for glioma treatment.

### Expression of pro-apoptotic protein Bim was down-regulated by SPHK1

**Figure 4:**
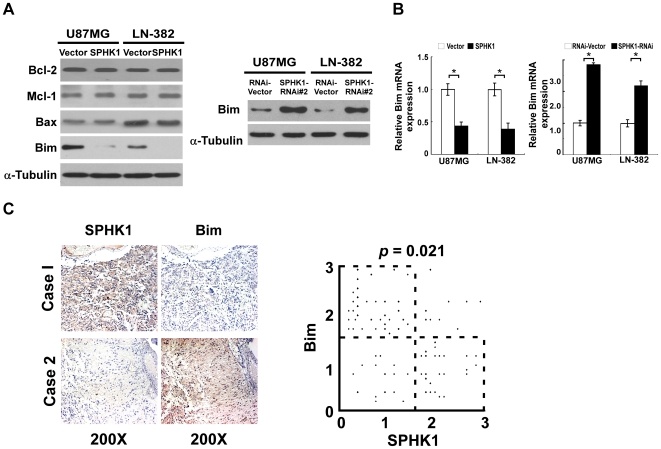
The expression of Bim is correlated with SPHK1 *in vitro* and *in vivo*. (A) The expression of BimEL protein level was significantly decreased in SPHK1 overexpression cells (left panel) and increased in SPHK1 knocked-down cells (right panel). (B) Overexpression of SPHK1 significantly decreased mRNA level of Bim (left panel), while knockdown of SPHK1 increased transcriptional level of Bim (right panel). (C) Examples of Bim expression in correlation with SPHK1 in human primary glioma specimens. Left panel, IHC staining of SPHK1 and Bim in glioma. Right panel, correlation of Bim and SPHK1 (n = 82; *p* = 0.021). Each data point represents one glioma specimen, and 24 of 33 (72.7%) glioma specimens with high SPHK1 expression displayed low expression of Bim, whereas 34 of 49 (69.4%) glioma specimens that showed low SPHK1 expression exhibited high expression of Bim. 0, no staining; 1, low staining; 2, moderate staining; 3, high staining.

Bcl-2 family proteins have been demonstrated to play a pivotal role in regulating and executing the intrinsic apoptotic pathways [24], [25]. Therefore, we further investigated whether upregulation of SPHK1 could affect the expression of Bcl-2 family proteins in glioma cells. As shown in Figure 4A, the expression of Bcl-2, Mcl-1 or Bax was not altered in the SPHK1-overexpressing cells, whereas the expression of BimEL was significantly decreased. The other two major Bim isoforms, BimL and BimS, were undetectable in the cell lines as similar to previous reports [26]. Real-time RT-PCR was performed and found a consistent decrease in Bim mRNA level in SPHK1-overexpressing cells as compared with the vector-control (Figure 4B). In contrast, downregulation of SPHK1 significantly increased Bim expression at both protein and transcription levels (Figures 4A and 4B).

To further delineate the correlation between SPHK1 and Bim expression in glioma, we next examined clinical primary glioma specimens for the expression of SPHK1 and Bim. Among a total of 82 tumor cases examined, high levels of SPHK1 expression were seen in 33 cases (40.2%), whereas 49 cases (59.8%) had low or undetectable levels of SPHK1 expression (Figure 4C). It is particularly noteworthy that 24 out of 33 (72.7%) glioma samples that exhibited high SPHK1 expression displayed low expression of Bim, in contrast to the high level Bim expression in 34 out of the 49 (69.4%) glioma samples with low SPHK1 expression (Figure 4C). Furthermore, Spearman correlation analysis showed that the correlation between SPHK1 and Bim expression was statistically significant (*p* = 0.021), suggesting a potential involvement of Bim downregulation in SPHK1-induced anti-apoptotic state in glioma.

### FOXO3a activity was altered in SPHK1 overexpression or downregulation glioma cells

**Figure 5:**
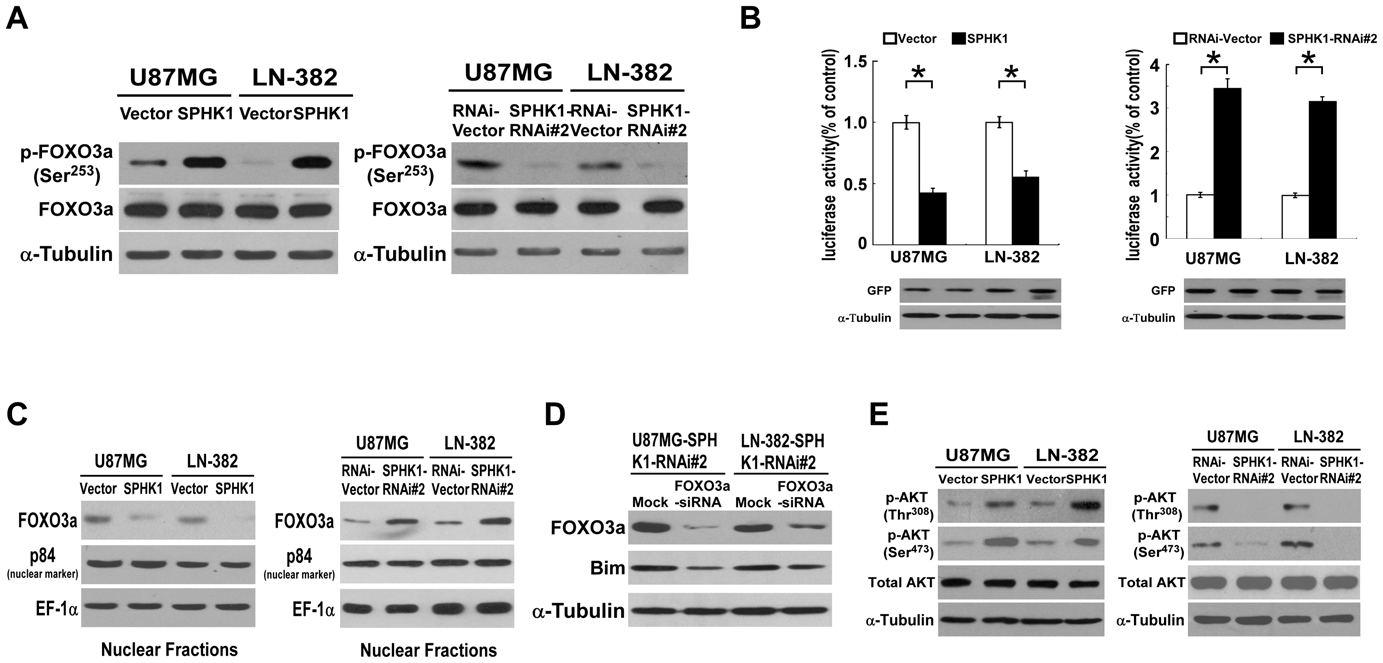
FOXO3a phosphorylation, transcriptional activity and Akt phosphorylation are regulated by SPHK1 in glioma cells. (A) SPHK1 phosphorylates FOXO3a in glioma cells. (B) FOXO3a-dependent transcription activity is regulated by overexpression (left panel) or knockdown (right panel) of SPHK1. Error bars represent mean ± SD from three independent experiments with similar results. *, *p*<0.05. The GFP expression was used to indicate the transfection efficiency. (C) WB analysis of nuclear FOXO3a protein in indicated cells. (D) SPHK1 knockdown-induced upregulation of Bim could be reversed by silencing of FOXO3a in indicated glioma cells. (E) The phosphorylation status of Akt at Thr^308^ and Ser^473^ were assessed by WB in SPHK1 overexpressed and knocked-down glioma cell lines.

To clarify the signal transduction pathways involved in regulating the expression of Bim in response to alterations of SPHK1 expression, the general expression level as well as activation status of transcription factor FOXO3a, a well known regulator of Bim [14], was examined in glioma cells with SPHK1 overexpressed or knocked down. As shown in Figure 5A, the SPHK1-overexpressed U87MG and LN-382 cells exhibited increased FOXO3a (Ser^253^) phosphorylation, in contrast, FOXO3a (Ser^253^) phosphorylation decreased dramatically in SPHK1-downregulated glioma cells. Furthermore, the luciferase activities of the FOXO3a reporter were examined to delineate the regulatory role of SPHK1 in FOXO3a transcriptional activity. As shown in Figure 5B, overexpression of SPHK1 indeed significantly reduced the activity of luciferase in the glioma cells, whereas the transcriptional activity of FOXO3a was elevated significantly in SPHK1 downregulated glioma cells, further suggesting that SPHK1 expression attenuated gene transcription driven by FOXO3a in glioma cells.

Since phosphorylation of FOXO3a was found to cause nuclear exclusion and consequently, inhibition of its transcriptional activity, we decided to examine whether SPHK1 modulated the nuclear expression of FOXO3a. WB analysis and cellular fractionation experiment showed that expression of FOXO3a in the nuclei was markedly increased in SPHK1 knocked-down cells and decreased in SPHK1 overexpressing cells (Figure 5C), suggesting that SPHK1 inhibited FOXO3a transcription activity through phosphorylation-dependent nuclear exclusion.

To further confirm the role of FOXO3a in regulating the expression of Bim in response to alterations of SPHK1 expression, we knocked down the expression of FOXO3a with specific siRNA in SPHK1 downregulated glioma cells. As shown in Figure 5D, SPHK1 knockdown-induced upregulation of Bim could be reversed by silencing FOXO3a, suggesting that FOXO3a is an important mediator of SPHK1-induced Bim expression.

### SPHK1 downregulated FOXO3a transcriptional activity via PI3K/Akt signaling

To elucidate the molecular mechanism via which SPHK1 regulates the activity of FOXO3a, the expression level and phosphorylated status of FOXO3a were examined in SPHK1-overexpressing and -knocked down glioma cells. Real time RT-PCR analysis revealed that SPHK1 did not change FOXO3a expression in transcriptional level (data not shown). WB analysis showed that the total expression level of FOXO3a did not change in glioma cells with SPHK1 overexpressed or knocked down, whereas the phosphorylation level of FOXO3a was significantly increased in SPHK1-transduced glioma cells as compared with vector-control cells, and a dramatic reduction of phosphorylation of FOXO3a was observed in SPHK1 knockdown cells (Figure 5A).

**Figure 6:**
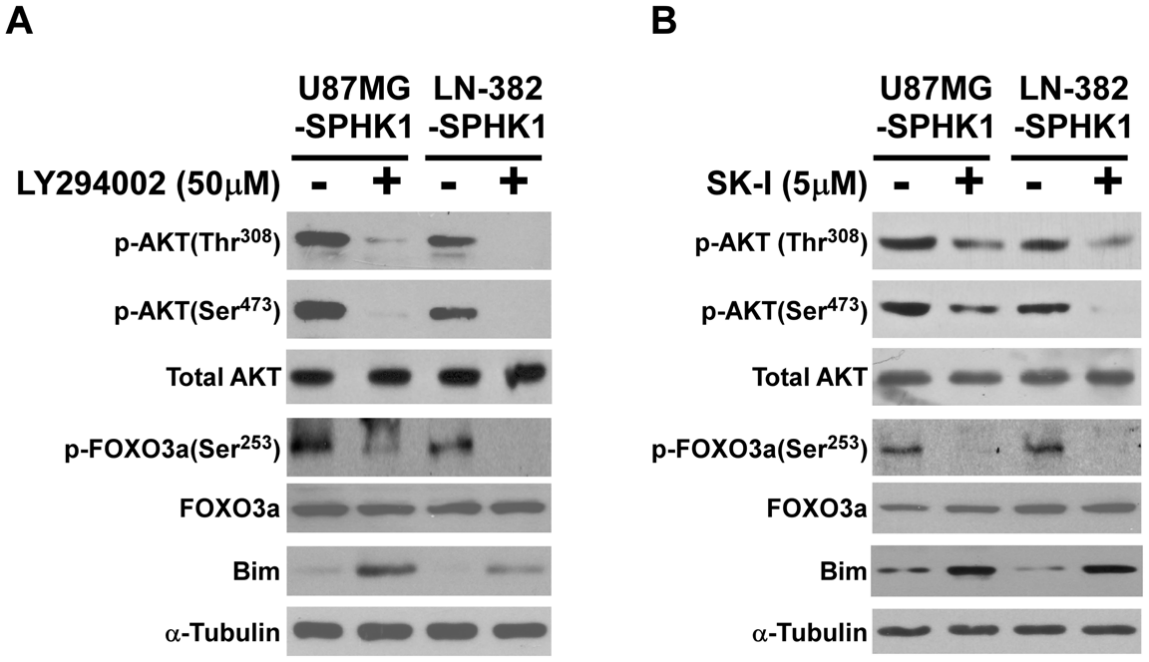
The effect of SPHK1 on Akt/FOXO3a/Bim pathway can be inhibited by PI3K inhibitor or SPHK1 inhibitor. U87MG-SPHK1 and LN-382-SPHK1 cells were incubated with 50 µM LY294002 (A) or 5 µM SK-I (B) for 24 h and cell lysates were harvested and subjected to WB. phospho-Akt (p-Akt), total Akt, p-FOXO3a, total FOXO3a and Bim were detected with specific antibodies, respectively.

It was reported that phosphorylation of FOXO3a could be mediated through activation of the Akt signaling pathway. Thus, we were promoted to further examine whether deregulation of SPHK1 could stimulate the activity of Akt kinase. As shown in Figure 5E, the phosphoryaltion level of Akt was upregulated by overexpression of SPHK1 and downregulated when SPHK1 was knocked down in U87MG and LN-382 glioma cells, suggesting a potential role of Akt in the signaling cascade that mediates FOXO3a phosphorylation/inactivation in response to SPHK1 upregulation. To further confirm this observation, we inhibited the PI3K activity by using its pharmacological inhibitor LY294002 and subsequently analyzed the levels of phosphorylated Akt, phosphorylated FOXO3a and Bim. As shown in Figure 6A, both phospho-Akt (Thr^308^ and Ser^473^) and phospho-FOXO3a (Ser^253^) were lowered after LY294002 treatment, while Bim protein level was increased significantly, suggesting that the effect of SPHK1 on FOXO3a phosphorylation was dependent on PI3K/Akt signaling.

### Inhibition of SPHK1 kinase activity abrogated the effects of SPHK1 on Akt/FOXO3a/Bim pathway in glioma cells

To further delineate the key role of SPHK1 in triggering the activation of Akt/FOXO3a/Bim signaling cascade, the kinase activity of SPHK1 was suppressed by SPHK1 inhibitor (SK-I) in SPHK1-overexpressing glioma cells. As shown in Figure 6B, SPHK1 inhibition decreased phosphorylation of Akt and FOXO3a and upregulated Bim, an overall response similar to RNAi knockdown of SPHK1. Taken together, this result, in conjunction with other data obtained in the rest of this study, identified SPHK1 as a trigger of the Akt/FOXO3a/Bim cascade.

## Discussion

The key finding of our present study is represented by the identification of a new chain of kinase reaction in glioma cells, activated by upregulated SPHK1, sequentially causing phosphorylated activation of Akt, phosphorylated inactivation of FOXO3a and downregulation of Bim, and consequently leading to evasion of glioma cells from programmed cell death or abrogation of chemotherapeutic agents-induced apoptosis.

Evasion of apoptosis is a hallmark of most human tumors and constitutes an important clinical problem. Many therapeutic agents act against cancers through inducing apoptosis, and resistance to cancer therapies usually involves intrinsic or extrinsic abrogation of the apoptotic mechanism in cancer cells. Accumulating evidences have indicated that SPHK1 could protect cancer cells from apoptosis [27]. Therefore, understanding the molecular mechanisms that mediate the anti-apoptotic effect of SPHK1 may lay a foundation for new anti-cancer strategies. To this end, the present study has for the first time provided a molecular dissection of an SPHK1-activated signal transduction cascade that leads to down-regulation of Bim and consequent resistance to apoptosis.

Bim, a member of Bcl-2 family, is a pro-apoptotic protein characterized by containing a single Bcl-2 homology 3 domain. In line with previous studies [28], [29], the expression of Bim appears to be under the regulation of SPHK1in glioma cells. Furthermore, overexpression of SPHK1 correlated with downregulation of Bim in clinical glioma samples. Interestingly, induction of Bim expression has also been linked to apoptosis induced by chemotherapeutic agents such as lovastatin, anisomycin-caused ribotoxic stress, or nitric oxide [30]–[32]. Thus, our finding provides a new insight into a possible mechanism that underlies SPHK1 induced resistance of glioma cells to apoptosis.

It is of note that regulation of Bim protein is attributed to transcriptional and posttranslational mechanisms [33]. One of the transcription factors involved in modulation the expression of Bim is a forkhead transcription factor, FOXO3a. Consistent with previous findings [15], [34]–[36], our study identifies FOXO3a as a mediator of SPHK1-associated downregulation of Bim, based on the following observations. Firstly, SPHK1 overexpression caused FOXO3a phosphorylation, and SPHK1 knockdown caused FOXO3a dephosphorylation in glioma cells. Secondly, pharmacological inhibitor of SPHK1 suppressed FOXO3a phosphorylation and increased Bim level. Thirdly, the transcriptional activity of FOXO3a was regulated by the level of SPHK1 protein in glioma cells.

It is evident that FOXO3a is under the regulation of the PI3K/Akt pathway [37]. Akt seems to be essential in phosphorylating FOXO3a in current study because our results showed activation of Akt and Ser^253^ phosphorylation of FOXO3a in SPHK1-overexpressing glioma cells, as well as Akt inactivation and Ser^253^ dephosphorylation of FOXO3a in SPHK1-downregulated glioma cells. The results of our study using LY249002 to suppress PI3K further confirmed that activation of FOXO3a was attributable to deregulation of PI3K/Akt signaling. Thus, data obtained from the present investigation indicate that PI3K/Akt signaling may play a role in mediating the regulation of FOXO3a by SPHK1. Furthermore, studies have demonstrated that SPHK1 can activate the PI3K/Akt pathway in human cancers [19], [38]–[42]. In glioma cells, as shown by our study, upregulated SPHK1 activates PI3K/Akt signaling and supports cell survival through modulation of FOXO3a and Bim, representing a new mechanism possibly underlying the development of glioma and probably, its resistance to pro-apoptotic therapeutics. Nevertheless, mechanism by which SPHK1 activates PI3K in glioma cells, i.e., via a direct versus indirect interaction between two molecules, remains to be determined. In this context, it is of note that sphingosine, sphingosine 1-phosphate (S1P) and ceramide are inter-convertible sphingolipids associated with numerous cell functions including proliferation, apoptosis and migration [43]. It has been demonstrated that Ceramide mediates and triggers cell growth arrest or apoptosis by inactivating PI3K/Akt [44], whereas S1P enhances proliferation and inhibits ceramide-mediated apoptosis [45], [46]. Furthermore, SPHK1 is a key regulator of the ceramide/S1P biostat since it is responsible for phosphorylating sphingosine, a catabolite of ceramide, to form S1P [43]. One may postulate that SPHK1-induced PI3K/Akt activation is attributable to decreased levels of sphingosine and ceramide. Alternatively, another possibility can be linked to extracellular S1P signaling via the S1P1 G protein-coupled receptor, which activates Rac, Fak and PI3K [47]. Further studies that aim at completely delineating the interaction between SPHK1 and PI3K/Akt will help develop new molecular targets for glioma therapy.

Treatment of glioma remains a clinical challenge. Currently available therapeutic strategies against glioma generally only delay local progression, and recurrence associated with resistance to therapies largely contributes to the high mortality of the disease. However, malignant glioma are typically infiltrative in nature. Thus, gaining new insights into the mediators of the apoptotic response in glioma will enable the development of novel anti-tumor strategies. In this context, targeting SPHK1 or its downstream signaling molecules as identified by our present study might represent new and potential strategies against human glioma.
